# Molecular epidemiology of *Megalocytivirus* in freshwater angelfish (*Pterophyllum scalare*) from Johor, Malaysia

**DOI:** 10.14202/vetworld.2023.2158-2172

**Published:** 2023-10-21

**Authors:** Che Azarulzaman Che Johan, Muhd Danish Daniel Abdullah, Sharifah Noor Emilia, Sandra Catherine Zainathan

**Affiliations:** 1 Department of Aquaculture, Faculty of Fisheries and Food Science, Universiti Malaysia Terengganu, 21030 Kuala Nerus, Terengganu, Malaysia; 2 Institute of Climate Adaptation and Marine Biotechnology, Universiti Malaysia Terengganu, 21030 Kuala Nerus, Terengganu, Malaysia

**Keywords:** epidemiology, genotype I, infectious spleen and kidney necrosis virus, *Megalocytivirus*, ornamental fish, risk factors

## Abstract

**Background and Aim:**

Malaysia has more than 630 culturists who are involved in the ornamental fish industry and culture 250 species, including local and exotic species. Among these viruses, megalocytiviruses have been associated with severe systemic diseases and economic losses in ornamental fish. The intensity of *Megalocytivirus* infection in *Pterophyllum scalare* in Malaysia remains unknown. Thus, this study aimed to investigate the occurrence of *Megalocytivirus* while discovering its associated risk factors and the genotypes of its causative agents in an ornamental fish farm in Malaysia.

**Materials and Methods:**

Seven broodstock pairs of *P. scalare* were used in this study to follow the life stages of fish, from egg to market size. Water samples and other samples, such as mucus swabs, gill swabs, *P. scalare* eggs, fries, juveniles, snails, snail eggs, live feed (Tubifex worms and *Moina* spp.), sediment samples, and wild fish, were collected periodically for initial environmental sampling from day 0 to day 60. Nested polymerase chain reaction amplifications were performed for megalocytivirus-related sequences. The phylogenetic tree, including the sampled causative agents of megalocytiviruses, was inferred from the major capsid protein genes of all known Iridoviridae species. Pearson’s correlation coefficients were calculated to determine the strength of the correlation between the presence of megalocytiviruses in *P. scalare* samples and the associated risk factors.

**Results:**

A total of 312 out of 935 pooled and individual samples tested positive for the presence of *Megalocytivirus*-related sequences, except snail eggs and wild fish (*Poecilia reticulata*). No clinical symptoms were observed in any fish samples. *Megalocytivirus*-associated viruses detected in water samples indicate horizontal transmission of the virus. All the nucleotide sequences found in this study had high nucleotide identities of 95%–99 % and were closely related to *Megalocytivirus* genotype I infectious spleen and kidney necrosis virus. Risk factors associated with *Megalocytivirus* include water temperature, dissolved oxygen (DO), pH, ammonia, nitrate, nitrite, and the life stages of *P. scalare*. High *Megalocytivirus* infection was detected when the water temperature, DO, and pH were high in *P. scalare*, high water temperature and nitrate in the water samples, and the same rate of *Megalocytivirus* infection in *P. scalare* fry and juveniles.

**Conclusion:**

This is the first study to confirm the existence of different possible routes of megalocytivirus distribution in ornamental fish farms in Malaysia. Nevertheless, the connection between the mode of transmission and the risk factors for this virus needs to be explored further to recognize the evolution and potential new host species.

## Introduction

The ornamental fish industry has become popular and profitable because of its enormous economic interest in global trade and its aesthetic attractiveness [1]. The highest export percentage of ornamental fish is from Asia (>65%), and the three largest markets for the ornamental fish industry are USA, Japan, and Europe [2]. The most popular ornamental fish traded around the world are neon tetra, angelfish, goldfish, danios, and discus [3]. The guppy and neon tetra species alone account for more than 25% of the market in volume and more than 14% in value [4]. The global ornamental fish industry includes more than 2500 species, including 60% freshwater and 40% of marine species [3]. Since the 1970s, the industry has increased by 14% in the production of neon tetras, angels, goldfish, danios, discus, guppy, and zebra danio [5]. The industry depends mostly on captive-bred freshwater fish and a substantial quantity of wild-caught fish and crustaceans [6]. Malaysia was one of the top ten countries with the highest export value in 2016, with global exports of US$14.09 million [3]. According to Malaysia’s annual fisheries data, the values for ornamental fish imports and exports in 2017 were RM 4,342,143,046 and RM 3,157,650,332, respectively [7].

However, ornamental fish have been associated with viral diseases, including *Megalocytivirus* infections. *Megalocytivirus* causes high mortality and acute infections in many marine and freshwater fish species, including freshwater ornamental fish [8]. In general, megalocytivirus infection causes non-specific gross symptoms similar to many other diseases, such as lethargy, loss of appetite, skin darkening, distended body cavity, and pale gills [8]. Megalocytiviruses are large linear single-dsDNA genomes [9] with large icosahedral DNA measuring 120–200 nm in diameter [10]. Initially, megalocytiviruses were divided into three major groups, including infectious spleen and kidney necrosis virus (ISKNV), red sea bream iridovirus (RSIV), and turbot reddish body iridovirus [8] based on genetic sequencing. Based on the geographic locations and genetic variations of major capsid protein (MCP) genes, 48 Asian and Australian *Megalocytivirus* isolates were grouped into three different clusters: Genotype I was isolated from several Asian countries, genotype II was from freshwater fish infected with megalocytiviruses from Asia and Australia, and genotype III originated from Chinese and Korean flatfish megalocytiviruses [8]. Recently, novel members have been grouped into the genus *Megalocytivirus* including scale drop disease virus isolated from the Southeast Asian *Lates calcarifer* [11] and European chub iridovirus isolated from the European chub (*Squalius cephalus*) [12]. Disease outbreaks due to *Megalocytivirus* have been observed in freshwater ornamental fish species such as *Trichopodus leeri*, *Trichopodus microlepis*, *Colisa lalia, Xiphophorus maculatus, Xiphophorus helleri, Poecilia sphenops*, *Lebistes reticulatus*, *Astronotus ocellatus*, *Hyphessobrycon innesi*, *Ptetrophyllum eimekei, Pterophyllum altum*, and *Danio rerio* (reviewed in Johan and Zainathan [8]). Malaysian freshwater fish species are positive for *Megalocytivirus*-associated viral infections, including *Apistogramma ramirezi*, *Xiphophorus hellerii*, *X. maculatus*, *P. sphenops*, *Trichopodus trichopterus* [13], and *D. rerio* and *Trichogaster lalius* [14]. Ornamental fish in other Asian countries, such as Singapore, Indonesia, and Thailand [8], are also affected by megalocytiviruses. Juvenile ornamental fish are the most susceptible to *Megalocytivirus* infections [8].

Common clinical signs in ornamental fish include lethargy, skin lesions, uncoordinated swimming, lack of appetite, distended and pale bodies, pale gills, hemorrhages, and ulceration [8]. To the best of our knowledge, the risk factors associated with *Megalocytivirus* infection in ornamental fish have not yet been reported. However, high water temperatures are known to increase *Megalocytivirus* [8]. Mortality outbreaks in tropical ornamental fish that are constantly maintained at high water temperatures have been detected throughout the year [15], irrespective of the month or season. The survivability of *Megalocytivirus* ranges from 20°C to 32°C [8]. The Australian Department of Agriculture reported that the infectivity of *Megalocytivirus* is inversely proportional to time and temperature [16]. Infectious spleen and kidney necrosis virus can remain infective even when isolated and kept under −70°C for >8 months and 40°C for 30 min, but the virus was inactivated when it was isolated and kept under 50°C for 30 min [16]. Therefore, *Megalocytivirus* can remain active throughout the year at optimum temperatures and contribute to the occurrence of disease outbreaks [17]. Few studies have reported the effect of temperature on *Megalocytivirus*, specifically in ornamental fish [8]. Other risk factors contributing to *Megalocytivirus* transmission in ornamental fish include transportation [18], life stage, and translocation [8]. Genetic variations exist within the genus *Megalocytivirus* [19]. Based on the phylogenetic tree generated by [20], two clusters were found within the genus *Megalocytivirus*. Cluster I included ISKNV and Cluster II mainly included RBIV, and orange-spotted grouper iridovirus, OSGIV. Sequencing analysis of *Megalocytivirus* MCP revealed that the ISKNV strains in other studies conducted in Malaysia demonstrated high nucleotide identity with each other and reference ISKNV: 97%–100% [13], 96%–100% [21], and 98%–100% [14]. Based on the phylogenetic tree, ISKNV strains are closely related to ISKNV and can be classified as *Megalocytivirus* genotype I [13, 14, 21]. In other countries, 24 species of ornamental fish from Brazil were found to be *Megalocytivirus*-positive by polymerase chain reaction (PCR) [22]. The positive samples demonstrated 99%–100% similarity with *Megalocytivirus* isolate Sabah/RAA1/2012 (JQ253374) from Malaysia. The first report of *Megalocytivirus* infection in India was reported in 10 ornamental fish species in India [23]. The sequence and phylogenetic analyses revealed that all positive samples were highly similar (98%–100%) to ISKNV strains from Malaysia, Japan, and Australia. Hence, it is vital to study the genetic variation in megalocytivirus strains, as they have the potential to produce new strains that may have novel properties and enhanced pathogenicity.

This study aimed to determine the presence or absence of *Megalocytivirus* and to determine the genotype of *Megalocytivirus* strain ISKNV in Malaysian *Pterophyllum scalare*. In addition, an epidemiological study to detect *Megalocytivirus* in freshwater angelfish (*P. scalare*) may provide new information on the risk factors and transmission routes of *Megalocytivirus* in Malaysia. Thus, it could minimize *Megalocytivirus* infections in Malaysian ornamental fish farms. This study has the potential to reduce the possible risks posed by these viral particles in terms of their spread and transmission to other fish species. Therefore, determining associated risk factors and genome characterization is a new approach for discovering new genotypes/strains of *Megalocytivirus* and new isolates based on sequence data. This represents a landmark epidemiological study of *Megalocytivirus* infections in freshwater angelfish in Malaysia.

This study aimed to detect and analyze megalocytiviruses isolated from its causative agents of freshwater angelfish (*P. scalare*) in Malaysia. This study also revealed the first possible routes of detection of the Malaysian *Megalocytivirus* strain ISKNV and the risk factors associated with viral disease in ornamental fish farms in Malaysia.

## Materials and Methods

### Ethical approval

No live fish were used in this study. All the samples and target organs examined were collected from a fish farm and subjected to laboratory analyses to detect *Megalocytivirus* and its associated viruses.

### Study period and location

This study was conducted from March 2019 to January 2020 at an ornamental fish farm in Johor, Southern Malaysia.

### Sampling station and experimental design

A cross-sectional study was conducted at an ornamental fish farm in southern Malaysia to detect the presence of megalocytiviruses during a routine *P. scalare* production. Before this study, no attempt had been made to conduct an epidemiological study at ornamental fish farm in Malaysia. To investigate the risk factors associated with megalocytiviruses on *P. scalare* farms, all breeding processes and hatchery management followed farm routines from the beginning until day 60 to investigate the risk factors associated with megalocytiviruses on the *P. scalare* farm. Before sampling, epidemiological data, including risk factors and the nature of disease outbreaks, were obtained from the farm. The risk factors associated with *Megalocytivirus* infections and diseases, such as fish age, water system, live feed, life stage (egg to juvenile), and water quality parameters, were evaluated during each sampling period. All the clinical signs associated with megalocytiviruses were observed and recorded during sampling. Three tubes of *P. scalare* eggs on day 0 (no pool), 415 samples of *P. scalare* fry (n = 200 fry from day 10 in 40 pools, n = 155 fry on day 20 in 31 pools; and n = 60 fry on day 30 in 12 pools), and 100 individual samples of *P. scalare* juveniles (n = 50 juveniles on days 45 and 60) were collected during the study period (Table-1). Information related to farm management was collected over 1 year of routine sampling. Basic data on the hatchery operating systems, such as the water system, hatchery plan, type of species cultured, history of disease outbreaks, and husbandry practices, were obtained before sampling.

**Table-1:** Details of experimental design including age of *Pterophyllum scalare* (days), sample collection type, and number of samples collected during sampling.

Days	Samples collected	Vol./No. of samples	Pool (OIE, 2019)	Size	Isolated organs
0	Water samples	500 mL	1 replicate		
	Fish eggs	3 tubes	No pool		Whole eggs
	Tubifex worms	50 g			
	Sediments	700 g			
10	Water samples	500 mL	1 replicate		
	Fry	200	5	<2 mm	Whole body
	*Moina*spp.	50 mL			
	Sediments	700 g			
20	Water samples	500 mL	1 replicate		
	Fry	155	5	~4–6 mm	Whole body
	Tubifex worms	50 g			
	Sediments	700 g			
30	Water samples	500 mL	1 replicate		
	Fry	60	5	~7–8 mm	Whole body
	Tubifex worms	50 g			
	Sediments	700 g			
45	Water samples	500 mL	1 replicate		
	Juvenile	50	No pool	~10–12 mm	Whole body
	Tubifex worms	50 g			
	Sediments	700 g			
60	Water samples	500 mL	1 replicate		
	Juvenile	50	No pool	~15–20 mm	Whole body
	Tubifex worms	50 g			
	Sediments	700 g			

A natural breeding technique was applied to produce new offspring of *P. scalare* at the farm. Initial environmental sampling (Table-2) was conducted at an early stage of the study to detect the presence of *Megalocytivirus*-related sequences in environmental samples and to obtain baseline data of positive *Megalocytivirus*-related sequences in such samples. All initial environmental samples, such as water samples from the inlet river and well, outlet, broodstock pond, and breeding pail; sediment from the broodstock pond, grow-out pond, river and outlet, broodstock gill swab, broodstock mucus swab, wild fish from the river, live feed (*Tubifex* worms and *Moina* spp.), snails, snail eggs, and water quality parameters, such as nitrate (NO^3-^), nitrite (NO^2-^), ammonia (NH_3_), pH, dissolved oxygen (DO), temperature, and salinity, were taken before the breeding of *P. scalare*.

**Table-2:** Fresh samples collected during initial environmental sampling at the farm in Johor, Malaysia.

Type of sample	Number of samples/pcs
Water	
River	1
Well	1
Broodstock pond	1
Breeding pails	1
Outlet	1
Mucus swab	14
Gill swab	14
Live feed	
Tubifex worms	3
Sediment	
River	3
Broodstock pond	3
Grow-out pond	3
Outlet	3
Snails	10
Snail eggs	1
Wild fishes (River)	9

Sterile wooden cotton-tipped swabs were prepared to swab the gills and mucus from the broodstock of *P. scalare* and collect the mucus from the gills and body of the fish. Gill and mucus samples were collected to evaluate a non-invasive method for detecting *Megalocytivirus*-related DNA in *P. scalare*. Both sides of the fish gills and the whole fish body (from the head to the caudal fin) were swabbed before placing the broodstocks in separate breeding pails. The cotton-tipped swab was placed and shaken vigorously in an associated tube filled with 3 mL viral transport media (VTM) containing 1X Hanks Balanced Salt solution and 2% fetal bovine serum.

Water samples from the inlet (river and well), outlet, broodstock pond, breeding pail, and grow-out pond from the initial environmental sampling and from day 0 to day 60 were collected directly using 500 mL sterile bottles to confirm the horizontal transmission of *Megalocytivirus* in water throughout the farm. In this study, possible vectors of *Megalocytivirus*-presence were collected. Snails (n = 10) and snail eggs (n = 1) were sampled using a sterile spoon by scraping them from the edge of the grow-out ponds and transferring them into 50 mL microcentrifuge tubes. Wild fish, such as *Betta kuehnei* (n = 2), *Poecilia reticulata* (n = 1), *Xenentodon cancila* (n = 3), and *Notropis hudsonius* (n = 3), were freshly caught from the river using a fish net and kept separately in 50 mL microcentrifuge tube.

Live feeds, such as *Tubifex* (~50 g) and *Moina* spp. (50 mL) (depending on the availability of the feed) used to feed *P. scalare* from the initial environmental sampling and day 0 until day 60 sampling were collected once early in the morning. Next, the sediment samples from the river, broodstock pond, grow-out pond (initial environmental sampling), days 20, 30, 45, and 60, and outlet (initial environmental sampling and day 0 until day 60) were collected (~700 g) using a sterile scooper that has been sanitized with 70% ethanol and transferred into zip lock bags. All the samples were kept in the −20°C freezer at the farm until day 60 of the sampling and then the samples were brought and kept in a −20°C freezer at Biosystem Fisheries Laboratory, Faculty of Fisheries and Food Science (FPSM), Universiti Malaysia Terengganu.

Water parameter data were evaluated at a depth of approximately 1 m in the river, well, *P. scalare* broodstock ponds, breeding pails, grow-out ponds, and outlet to examine *Megalocytivirus-*associated risk factors. Water quality parameters assessed included temperature, pH, DO content, and salinity. These parameters were measured *in situ* using handheld YSI™ water quality parameters (Pro Plus Jula YSI Pro 20 DO 550A TSS) and ammonia, nitrate, and nitrite’s concentration were assessed using a freshwater master test kit (Aquarium Pharmaceuticals, API). The collected data were used to determine the probable relationship between *Megalocytivirus* or its related viruses and water quality at various life stages of *P. scalare*.

### Sample processing

#### Fish sample processing

All samples were pooled and processed throughout the research period based on fish size [24]. The *P. scalare* samples were processed according to fish size (Table-1). Due to the small sample size, whole fish eggs (FE) and fish samples were cut and minced separately. The samples were homogenized using sterile scissors using a tissue homogenizer in a 50 mL tube containing 5 mL of VTM. The homogenized tissues were centrifuged for 5 min at 8000× *g*, and the supernatants were collected and stored at −20°C until the DNA extraction.

#### Processing of water, gill swab, mucus swab, live feed, snail, snail eggs, wild fish, and sediment

All water samples throughout this study were divided into six 50 mL sterile centrifuge tubes. Each water sample had a total volume of 300 mL. The water samples were centrifuged at 4°C for 30 min at the maximum speed. Approximately 50 μL supernatant of water sample from each tube was transferred into 1.5 mL microcentrifuge tube. The total final volume in each 1.5 mL microcentrifuge tube was 300 μL. The water sample supernatants were maintained at −20°C before DNA extraction. The gill and mucus swab samples were centrifuged at 4°C for 5 min at the maximum speed. Then, approximately 200 μL supernatants from each sample were transferred separately into 1.5 mL microcentrifuge tube and kept at −20°C. Live feeds (*Moina* spp. and *tubifex* worm) were cut and minced into small pieces using a sterile scissor in 1.5 mL centrifuge tubes containing 500 μL VTM. The samples were homogenized using a sterile tissue homogenizer. The homogenized live feed samples were then centrifuged for 5 min at 5000× *g*. Approximately 200 μL of supernatants from each sample were transferred into a new 1.5 mL microcentrifuge tube separately and kept in a −20°C freezer.

The snails (excluding the shell) and snail eggs were cut and minced using sterile scissors in a 1.5 mL microcentrifuge tube containing 500 μL of VTM. The samples were homogenized using a sterile tissue homogenizer. Then, the homogenized snails and snail eggs were centrifuged for 5 min at 5000× *g*. Approximately 200 μL of supernatants from each sample were transferred into a new 1.5 mL microcentrifuge tube separately and kept in at −20°C. The whole body of wild fish was cut and minced in 1.5 mL microcentrifuge tube containing 500 μL VTM. The samples were homogenized using a sterile tissue homogenizer and centrifuged at 5000× *g* for 5 min. Approximately 200 μL of supernatants from each sample were transferred into a new 1.5 mL microcentrifuge tube separately and stored in at −20°C prior to DNA extraction. Sediment samples from the initial environmental sampling and from day 0 to day 60 were homogenized using a sterile mortar and pestle. Then, the homogenized sediments were then transferred separately into a 15 mL centrifuge tube containing 5 mL VTM and centrifuged at 4°C for 30 min at maximum speed. Approximately 200 μL supernatants of each sediment sample were collected and transferred into a new 1.5 mL tube. The supernatants of sediment samples were stored at −20°C before DNA extraction. The GF-1 Viral Nucleic Acid Extraction Kit (Vivantis Technologies Sdn. Bhd., Selangor, Malaysia) was used to extract DNA [21] according to the manufacturer’s instructions.

### Nested PCR amplification

Throughout the study period, the extracted DNA samples were subjected to nested PCR amplification to detect the presence of *Megalocytivirus*-associated virus using a 2X MyTaq™ Mix Kit (Bioline, Selangor, Malaysia) following the manufacturer’s instructions. Nested PCR was performed according to the modified method described by Zainathan *et al*. [21]. The primers were based on forward primer C1105 (5′-GGGTTCATCGACATCTCCGCG-3′) and reverse primer C1106 (5′-AGGTCGCTGCGCATGCCAATC-3′) for the primary reaction. For the nested reaction, forward primer C1073 (5′-AATGCCGTGACCTACTTTGC-3′) and reverse primer C1074 (5′-GATCTTAACACGCAGCCACA-3′) were used. A PCR reaction mixture with a total volume of 25 μL was prepared as follows: 2X MyTaq™ Mix (12.5 μL), Rnase-free water (9.0 μL), C1105 (10 μM, 0.5 μL), C1106 (10 μM, 0.5 μL) and extracted DNA (2.5 μL) [21]. The amplification was performed using a T100™ Thermal Cycler (Bio-Rad, Selangor, Malaysia), which was programed to carry it out as follows: 10 min at 95°C and then followed by 95°C for 30 s, 55°C for 30 s, and 72°C for 1 min for 35 cycles. The final extension step was performed at 72°C for 5 min. The nested PCR amplification was performed with a total of 25 μL PCR mixture containing similar mixtures and the inclusion of C1073 (0.5 μL, 10 μM) and C1074 (0.5 μL, 10 μM) primers. The amplicon sizes of the primary and nested primers were 430 bp and 167 bp, respectively. The amplified PCR products from both reactions were then analyzed by electrophoresis, which was run for 45 min at 70V on 1.7% (w/v) agarose gel in tris-acetate-ethylenediaminetetraacetic acid buffer and stained with SYBR® Safe-DNA Gel Stain (Invitrogen, Waltham, Massachusetts, USA). An artificial positive control generated from the sequencing of *Megalocytivirus* Sabah (JQ253374.1) and field-positive sample was used as positive controls [13].

A total of 21 μL *Megalocytivirus*-positive nested PCR samples were delivered to First Base Laboratories Sdn. Bhd. (Selangor, Malaysia) for sequencing analysis. The phylogenetic analysis was based on DNA sequencing data. Sequences were compared with those in the GenBank database using BLASTn to obtain sequence identities. Sequences were then aligned with other *Megalocytivirus*-associated sequences using Clustal X2.0.12 (http://www.clustal.org/clustal2/) [25]: 20 *Megalocytivirus* MCP gene sequences for the sister group [26] and 2 *Ranavirus* sequences for the outgroup [27, 28]. Finally, the Molecular Evolutionary Genetics Analysis X software (https://www.megasoftware.net/) [29] was used to generate the phylogenetic tree after phylogenetic evaluation based on the coat protein genes of the samples and closely identical published sequences of other viruses (Pennsylvania State University, USA). Evolutionary distances were calculated using 1000 bootstrap replicates and the neighbor-joining method with the LG + F substitution model.

### Statistical analysis

To determine the strength of their correlations with the presence of *Megalocytivirus* in *P. scalare* samples, epidemiological data, including fish age, vectors, and water quality parameter values, were computed using Pearson’s correlation coefficient (r) values (statistical package for the social sciences statistics software version 20, IBM, USA). For this study, r values might vary between −1 and 1, with positive r values indicating a positive linear correlation and negative r values indicating a negative linear correlation. To determine the strength of the associations between variables, r values ranging from 0.00 to 0.39 were regarded as weak correlations, 0.40–0.59 indicated moderate correlations, and 0.60–1.0 indicated significant correlations. For the association to be statistically significant, the p-value must be < 0.05 [30].

## Results

### Hatchery management

Throughout the research period, appropriate hatchery management (Table-3) procedures such as adequate sanitation, proper husbandry techniques, a clean work environment, equipment maintenance, feed quality, and quarantine areas were disregarded at the hatchery site. Unattended biosecurity at farm facilities has also occurred. The river (temperature = 26.2°C, salinity = 0 ppt, DO = 6.72 mg/L, and pH = 7.2) and well water (temperature = 22.1°C, salinity = 0 ppt, DO = 7.52 mg/L, and pH = 7.6) were directly used in the farm. Furthermore, the outlet water was not properly filtered before being discharged from the farm. In addition, the eggs of *P. scalare* were maintained in breeding pails without being changed until they hatched and became fry (day 10). The fry was subsequently moved to a grow-out pond and cultivated to a marketable size (typically on days 45 and/or 60). *Moina* spp. or Tubifex worms were fed *P. scalare* twice daily, without an exact amount. The life feeds purchased from suppliers did not pass quality inspection but were applied directly to the fish. The breeding and grow-out ponds were shaded with black shading nets to avoid direct sunlight. Furthermore, no hygiene standards have been implemented for hatchery workers. No foot baths or handwashing stations were set up at the entry or exit points of the farm. Many farm equipments are not adequately disinfected or stored.

**Table-3:** Management of the farm’s hatcheries and husbandry procedures.

Aspects	Description
Staffs’ attires and tools	• Different areas of the hatchery employed similar equipment.
	• The feeding tools are not decontaminated on a regular basis and are not maintained in suitable, clean areas.
	• The clothing of hatchery employees was not sanitised before prior to entry to the hatchery sites.
Decontamination	• Prior to feeding, water changes, and pond cleaning, the farmers do not use disinfection procedures.
Animal carrying	• The farm does not use particular stocking density.
Nurturing condition	• The *P. scalare*was cultured at water salinity of 0 ppt and temperature ranging from 29.3°C to 32.5°C.
	• There is no monitoring of water parameters at the breeding area.
Water system	• Lack of UV sterilization and insufficient filtration of the hatchery’s incoming water supply (mainly sand and sediment filter).
	• The water from the broodstock pond is reused at the breeding area.
Outlet water	• The water was not treated adequately before it exits the farm.
Live feed	• There was no feed quality testing prior to feeding.
Feeding rate	• There was no precise amount or frequency of feeding rate; the *P. scalare*was fed 2 times per day.
Fish shelter	• There was no fish shelter for the small fish to hide from the larger fish.
Security in the hatchery	• The dogs were trained to patrol the hatchery area; the dogs merely enter the fish ponds on a daily basis.
Open facility	• The egg, fry, juvenile and broodstock of *P. scalare*were reared in plastic pail and concrete pond (open facility).
Change of water	• The water was not changed from the time the eggs were laid until the fry were transported to the grow-out pond.
	• Water is changed twice a day throughout the juvenile stages.

UV=Ultraviolet, *P.* *scalare=Pterophyllum scalare*

To date, there have been no epidemiological assessments of *Megalocytivirus* in Malaysian ornamental fish farms. However, several studies have reported the presence of *Megalocytivirus* DNA in various varieties of ornamental fish in Malaysia. The positive results from the PCR analysis of the samples also corroborated the initial environmental sampling findings of this study in that there were additional factors that caused the outbreak of *Megalocytivirus* in the ornamental fish farm. The results revealed the presence of *Megalocytivirus* before the most recent sampling at the ornamental fish farm.

### Water quality parameters

Before sampling, the average water parameters of the initial environmental sampling (river, well, broodstock pond, breeding pail, and outlet) and current sampling (days 0–60) were measured, and are presented in Table-4 [31, 32]. The normal range of water quality parameters for freshwater fish farms was based on previous studies by Trivedi and Dubey [31] and Devi *et al*. [32]. The temperatures of all the water samples were within the normal range during sampling. However, the water temperature of the well (22.1°C) during initial environmental sampling and from day 10 until day 60 (21.9°C–22.9°C) was below the normal range of water temperature (25°C–32°C) due to rainy season [33]. The DO concentration for the broodstock pond, breeding pail, and days 0, 10, 20, 30, 45, and 60 was within the standard range of DO concentration (>5.00 mg/L). The salinity of all the water samples (0 ppt) prior to sampling was at the expected value (<0.5 ppt). In addition, the pH values of all water parameters were between 6.4 and 7.6. The normal range of pH for aquaculture freshwater fish is from 7.5 to 9.0 [31]. The pH values of the river (7.2), outlet (6.4), day 0 (7.0), and day 10 (6.9) were below normal ranges. As for the nitrate concentration, the water sample on day 10 demonstrated the highest reading (16 mg/L), which was above the normal range (0.2–10 mg/L) [32]. The nitrite concentration of all water samples (0.00–0.34 mg/L) was within the normal range (0.00–0.50 mg/L) [31]. In addition, the ammonia concentration of water sample for broodstock pond, outlet, day 0, and day 10 was observed to be higher than the average range (0–0.10 mg/L) [31]. Salinity and nitrite concentrations were within normal ranges [31] throughout the sampling period.

**Table-4:** The measurement of water quality parameters throughout the study.

Parameters	Initial environmental sampling					Current sampling/Day						Normal range	Reference
	
	River	Well	Broodstock pond	Breeding pail	Outlet	0	10	20	30	45	60		
Dissolved oxygen (mg/L)	6.72	7.52	3.79	3.60	3.80	1.53	1.76	2.06	1.96	1.96	1.98	>5.00	[31]
pH	7.2	7.6	7.6	7.6	6.4	7.0	6.9	7.5	7.5	7.6	7.6	7.5–9.0	
Salinity (ppt)	0.0	0.0	0.0	0.0	0.0	0.0	0.0	0.0	0.0	0.0	0.0	<0.5	
Temperature (°C)	26.2	22.1	29.2	28.6	28.9	26.2	22.6	22.9	21.9	22.6	22.5	25–32	
Ammonia (mg/L)	0.00	0.00	0.43	0.07	0.25	2.00	1.29	0.04	0.00	0.00	0.00	0–0.10	
Nitrite (mg/L)	0.00	0.00	0.00	0.00	0.00	0.00	0.34	0.00	0.00	0.00	0.00	0.00–0.50	
Nitrate (mg/L)	0	0	2	1	10	4	16	0	0	0	0	0.2–10	[32]

### Clinical signs

None of the fish samples showed gross symptoms during the study. Because of their small size, external and internal clinical symptoms could not be examined during the fry stage (10, 20, and 30 dph).

### Nested PCR

A total of 312 out of 935 samples were positive for *Megalocytivirus*-associated virus, except snail eggs (n = 3) and wild fish (*P. reticulata*) (n = 3) in the nested PCR (Table-5). From day 0 to 60, all freshly caught fish samples showed evidence of *Megalocytivirus* DNA. The positive result for FE (n = 3) on day 0 indicated the early detection of *Megalocytivirus* infection in *P. scalare*. Moreover, water samples from the inlet river (n = 4), inlet well (n = 32), broodstock ponds (n = 6), breeding pails (n = 20), and grow-out ponds (n = 20) tested positive for *Megalocytivirus* in this study. Plus, *Moina* spp. (n = 19), tubifex worm (n = 50), sediment from the river (n = 3), broodstock pond (n = 24), grow-out pond (n = 99), outlet (n = 69), snail (n = 10), wild fishes *B. kuehnei* (n = 2), *X. cancila* (n = 3), and *N. hudsonius* (n = 3) demonstrated *Megalocytivirus*-positive in the nested PCR. Positive swab samples of gills (n = 7) and mucus (n = 7) of *P. scalare* broodstock showed that this non-invasive method could be applied to detect *Megalocytivirus* DNA at the farm using nested PCR analysis. Based on the occurrence of *Megalocytivirus* (%) (Table-5), all fish stages of *P. scalare* were infected with *Megalocytivirus*. However, the highest occurrence of *Megalocytivirus* was observed on day 0 (100%).

**Table-5:** The total number of positive bands obtained from initial environmental and current sampling.

Samples	Nested PCR	Occurrence of *Megalocytivirus*(%)
Water		
Inlet	2/4	50
River		
Well	11/32	34
Broodstock pond	3/6	50
Breeding pail	8/20	40
Grow-out pond	7/20	35
Outlet	10/23	43
Swab		
Mucus		
Male	2/7	29
Female	4/7	57
Gill	4/7	57
Male		
Female	2/7	29
Fish		
Egg	3/3	100
Day 10	20/200	10
Day 20	55/155	35
Day 30	10/60	17
Day 45	26/50	52
Day 60	29/50	58
Sediment		
Inlet	2/3	67
River		
Broodstock pond	9/24	38
Grow-out pond	40/99	40
Outlet	28/69	41
Live feed		
*Tubifex*worm	26/50	52
*Moina*spp.	3/19	16
Snail	2/10	20
Snail egg	0/1	0
Wild fish		
*Betta kuehnei*	1/2	50
*Poecilia reticulata*	0/1	0
*Xenentodon cancila*	3/3	100
*Notropis hudsonius*	2/3	67
Total	312/935	

PCR: Polymerase chain reaction

### Sequencing and phylogenetic analysis

All 10 *Megalocytivirus*-positive samples from each type of samples including snails, Live feed *Moina* spp. (LFM), tubifex worms (LFTW), outlet sediment, river sediment, FE, *P. scalare* fries at day 10 (FD10), fries at day 20 (FD20), fries at day 30 (FD30), and *P. scalare* juvenile at day 45 (FD45) collected from initial environmental and current samples were sent for sequencing analysis. Other positive samples were not sent for sequencing analysis because of the low intensity of the positive bands. In this study, multiple alignments of the nucleotide sequences identified that the samples collected from the initial environmental sampling and the current study (day 0–day 60) were from a similar member of the *Megalocytivirus* strain, ISKNV.

**Figure-1:**
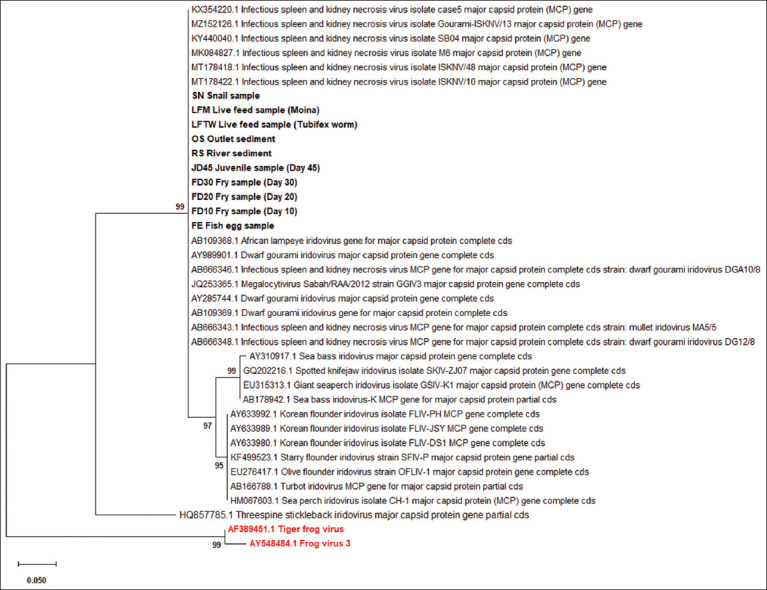
The phylogenetic tree was constructed for 167bp segments based on MCP gene sequences of ISKNV detected in 10 of Megalocytivirus-positive samples from this study (black bold) and 6 reference viruses from the genus Megalocytivirus using the neighbour-joining method and 1000 bootstrap replicates. JQ253365.1 Megalocytivirus Sabah/RAA/2012 strain GGIV3 major capsid protein gene. FLIV-PH MCP gene (AY633992.1), FLIV-JSY MCP gene (AY633989.1) FLIV-DS1 MCP gene (AY633980.1), ISKNV MCP gene for major capsid protein, strain: DGA10/8 (AB666346.1), Sea bass iridovirus-K MCP gene for major capsid protein (AB178942.1), SFIV-P major capsid protein gene (KF499523.1), DGIV major capsid protein gene (AY989901.1), OFLIV-1 major capsid protein gene (EU276417.1), GSIV-K1 major capsid protein (MCP) gene (EU315313.1), Threespine stickleback iridovirus major capsid protein gene (HQ857785.1), Turbot iridovirus MCP gene for major capsid protein (AB166788.1), Sea perch iridovirus isolate CH-1 major capsid protein (MCP) gene (HM067603.1), ISKNV MCP gene for major capsid protein, strain: mullet iridovirus MA5/5 (AB666343.1), ISKNV MCP gene for major capsid protein, strain: DGIV DG12/8 (AB666348.1), ALIV gene for major capsid protein (AB109368.1), DGIV gene for major capsid protein (AB109369.1), DGIV major capsid protein gene (AY285744.1), Sea bass iridovirus major capsid protein gene (AY310917.1) and Spotted knifejaw iridovirus isolate SKIV-ZJ07 major capsid protein gene (GQ202216.1) were included as sister group. Tiger frog virus (AF 389451.1) and Frog virus 3 (AY 548484.1) (red bold) were included as outgroup. The scale bar indicated distance values, the GenBank accession numbers were presented next to the virus species/strains, and the bootstrap values (%) were demonstrated next to the clades. The tree is drawn to scale (0.050), with branch lengths measured in the number of substitutions per site.

The *Megalocytivirus*-positive samples demonstrated similarities ranging from 95 to 99% to the MCP gene of ISKNV/10 from Oscar (India isolate) (MT178422.1), ISKNV/48 from Oscar (India isolate) (MT178418.1), MK084827.1 (ISKNV isolate M6 from molly in India), KY440040.1 (ISKNV isolate SB04 from *L. calcarifer* in Vietnam), KX354220.1 (ISKNV isolate case5 from *P. scalare* in Australia), and MZ152126.1 (ISKNV isolate Gourami-ISKNV/13 from giant gourami in India). The highest sequence similarity (99%) was obtained from sediment samples from outlet and snail samples. The assembled nucleotide sequences from this study were compared with representative *Megalocytivirus* sequences to determine the percentage identity between them. The sequence variations were demonstrated among the positive samples and between the BLAST results based on the pairwise sequence comparison of nucleotide sequence MCP gene (95%–100%) (Table-6). Phylogenetic analysis was performed to determine the genetic relationships between the *Megalocytivirus*-associated viral isolates collected from the positive samples in this study. Based on the phylogenetic tree, the samples were classified as ISKNV strain (Figure-1). The following two outgroups: AF389451.1 (Tiger frog virus) and AY548484.1 Frog virus 3 (FV3) from the genus *Ranavirus* and 20 sequences as a sister group from megalocytivirus MCP genes were included in the phylogenetic analysis. The phylogenetic tree provided evidence that the positive samples in this study came from freshwater fish infected with *Megalocytivirus* and belonged to the same genotype, known as genotype I ISKNV, from Asian countries.

**Table-6:** Pairwise sequence comparison of nucleotide sequence major capsid protein (MCP) gene similarities among nucleotide sequences of positive samples in this study and BLAST result with the MCP gene (published sequences) retrieved from the GenBank. Data presented in percentage of similarities for 167bp. Sequence variation were observed in the samples.

	1	2	3	4	5	6	7	8	9	10	11	12	13	14	15	16
1		96	98	98	98	98	99	97	98	99	96	96	96	96	96	96
2			98	97	99	97	99	98	97	99	98	98	98	98	98	98
3				98	99	96	98	97	98	99	96	96	96	96	96	96
4					100	98	98	99	100	100	98	98	98	98	98	98
5						99	98	99	100	100	98	98	98	98	98	98
6							98	97	99	99	95	95	95	95	95	95
7								98	99	100	99	99	99	99	99	99
8									99	100	97	97	97	97	97	97
9										100	98	98	98	98	98	98
10											99	99	99	99	99	99
11												100	100	100	100	100
12													100	100	100	100
13														100	100	100
14															100	100
15																100
16																

1 = *fe/day0, 2 = *fd10/day10, 3=fd20/day20, 4=fd30/day30, 5 = *jd45/day45, 6 = *rs (initial environmental sample), 7=os/day10, 8 = *lftw/day60, 9=lfm/day20, 10 = *sn (initial environmental sample), 11=MT178422.1 ISKNV isolate ISKNV/10 MCP gene from Oscar, 12=MT178418.1 ISKNV isolate ISKNV/48 MCP gene from Oscar, 13=MK084827.1 ISKNV isolate M6 MCP gene from molly, 14=KY440040.1 ISKNV isolate SB04 MCP gene from *L. calcarifer*, 15=KX354220.1 ISKNV isolate case5 MCP gene from *P. scalare,* and 16=MZ152126.1 ISKNV isolated Gourami-ISKNV/13 MCP gene from giant gourami. FE=Fish eggs, FD10=Fry Day 10, FD20=Fry Day 20, FD30=Fry Day 30, JD45=Juvenile Day 45, RS=River sediment, OS=Outlet sediment, LFTW=Live feed Tubifex worm, LFM: Live feed *Moina* spp., SN=Snail, ISKNV=Infectious spleen and kidney necrosis virus, MCP=Major capsid protein, *P. scalare*=*Pterophyllum scalare*

### Epidemiological data analysis

Pearson’s correlation coefficient values were analyzed to determine whether there was a relationship between the presence of *Megalocytivirus* in *P. scalare* and different water quality parameters, fish size, and fish age (Table-7). The significant risk factors associated with the presence of *Megalocytivirus* included water temperature, DO, pH, nitrate, nitrite, ammonia, and the life stages of *P. scalare* (p < 0.01). Positive correlations were identified between the presence of *Megalocytivirus* in the fish samples and water temperature (r = 0.57), DO (r = 0.51), and pH (r = 0.57). These results indicate that *P. scalare* samples were more susceptible to *Megalocytivirus* at high temperatures, DO, and pH. Similarly, a higher number of water samples were positive for *Megalocytivirus* DNA at higher water temperatures (r = 0.51) and nitrate concentrations (r = 0.61). In contrast, a lower number of sediment samples were positive for the presence of *Megalocytivirus* DNA at higher water temperature (r = −0.40) and higher DO (r = −0.45). Negative relationships were observed both DO (r = −0.70) and pH (r = −0.47) and the number of water samples that were positive for *Megalocytivirus* DNA, indicating that a lower number of water samples were positive for *Megalocytivirus* at higher DO and pH. Low susceptibility to *Megalocytivirus* infection in fish samples was observed at high ammonia (r = −0.50), high nitrate (r = −0.47), and high nitrite (r = −0.32). The fry and juvenile stages of *P. scalare* showed the same rate of positive correlation with *Megalocytivirus* infection (r = 1.00), indicating that both fry and juveniles were susceptible to *Megalocytivirus*.

**Table-7:** Assessment results of association between the presence of *Megalocytivirus* in fish and water samples with regards to water parameters and fish age based on Pearson’s correlation coefficient analysis.

Risk factors	p-value	r-value
1. High temperature:		
• High rate of Megalocytivirus infection in water samples	<0.01	0.51
• High rate of Megalocytivirus infection in fish samples	<0.01	0.57
• Low rate of Megalocytivirus infection in sediment samples	<0.01	−0.40
2. High concentration of dissolved oxygen, DO:		
• Low rate of *Megalocytivirus*infection in water samples	< 0.01	- 0.70
• High rate of Megalocytivirus infection in fish samples	<0.01	0.51
• Low rate of Megalocytivirus infection in sediment samples	<0.01	- 0.45
3. High pH:		
• Water samples were less susceptible to Megalocytivirus infection.	<0.01	−0.47
• Fish samples were highly susceptible to Megalocytivirus infection.	<0.01	0.57
4. Concentration of ammonia: Decreased the influence of *Megalocytivirus*infection in fish samples.	<0.01	−0.50
5. High concentration of nitrate:		
• Increased the influence of Megalocytivirus infection in water.	<0.01	0.61
• Lower the influence of Megalocytivirus infection in fish.	<0.01	−0.47
6. High concentration of nitrite: Lower rate of *Megalocytivirus*infection in fish.	<0.01	−0.32
7. The same rate of *Megalocytivirus*infection recorded in fry and juvenile stage of *P. scalare*.	<0.01	1.00

*P. scalare*=*Pterophyllum scalare*

## Discussion

To the best of our knowledge, this is the first epidemiological study on *Megalocytivirus* infection in *P. scalare* in Malaysia. No clinical symptoms of *Megalocytivirus* infection were detected across the life stages (egg, 10–60 dph) of the fish. The presence of a viral agent that causes megalocytiviruses was detected in 312 of 935 pooled and individual samples, including *P. scalare* samples (broodstock, eggs, 10 dph–60 dph), water samples (river, well, broodstock ponds, breeding pails, grow-out ponds, and outlet), sediment samples, snail samples, live feeds (*Moina* spp. and *tubifex* worms), wild fish (*B. kuehnei*, *X. cancila*, and *N. hudsonius*), and gill and mucus swab samples. Positive water samples indicated horizontal transmission of *Megalocytivirus*. The *Megalocytivirus*-associated risk factors identified in this study included hatchery management, water quality parameters (temperature, DO, pH, nitrite, nitrite, and ammonia), water and sediment samples, live feed, snails, wild fish, and life stage. All the nucleotide sequences in this study revealed a high nucleotide identity of 95%–99% to each other, MCP gene of ISKNV/10 from Oscar (India isolate) (MT178422.1) and ISKNV/48 from Oscar (India isolate) (MT178418.1). Phylogenetic analysis of the MCP gene nucleotide sequence confirmed that the variation of *Megalocytivirus* existed in each *Megalocytivirus*-positive sample based on the type of sample and life stage.

In the present study, *Megalocytivirus*-positive *P. scalare* samples did not show any gross signs. Similar results have been reported in previous studies by Zainathan *et al*. [13], Zainathan *et al*. [14] and Zainathan *et al*. [21] on ornamental fish in Malaysia. *Megalocytivirus*-infected ornamental fish, including *X. maculatus*, *P. sphenops* [13], *P. reticulata*, *T. leeri*, and *A. ramirezi* [21] from Southern Malaysia, demonstrated the absence of clinical signs. However, some Malaysian ornamental fish such as *T. trichopterus* have pale gills and enlarged livers in *T. trichopterus*, distended abdomen in *X. hellerii* [13] and darkened body in *X. maculatus* [21]. Other countries have demonstrated similar results for infected freshwater ornamental fish, such as *P. scalare* and *Puntius titteye* from India [23], and *P. scalare*, *Moenkhausia costae*, *Hemiodopsis gracilis* from Brazil [22]. Until now, no specific clinical signs have been observed to indicate the *Megalocytivirus* infection as *Megalocytivirus* develops similar clinical signs similar to those of other diseases [22, 34]. In Malaysia, *Megalocytivirus* is known to be an asymptomatic carrier [13, 21, 35]; therefore, the virus does not cause any visible symptoms but can be transmitted to other fish [36].

A total of 312 (pooled and individual) samples were positive for *Megalocytivirus* DNA. In 2022, a study was published in Malaysia on the molecular detection of *Megalocytivirus* ISKNV strain in oviparous pet fish [14]. The results showed that six pooled fish specimens (n = 30) of *D. rerio* and *T. lalius* were positive for genotype I ISKNV, as tested by nested PCR. The previous studies by Zainathan *et al*. [13], Zainathan *et al*. [21], and Subramaniam *et al*. [35] conducted in Malaysia demonstrated that several ornamental fish species, including *X. helleri*, *X. maculatus*, *P. sphenops*, *T. trichopterus, P. reticulata*, *T. leeri*, and *A. ramirezi* tested positive for *Megalocytivirus* DNA using the same PCR method. Polymerase chain reaction assays are frequently used to detect the viral DNA and MCP genes of *Megalocytivirus* [37] and have been proven beneficial for early diagnosis [38]. There are many types of PCRs [9]. However, nested PCR is widely used to detect the presence of the DNA virus in many studies [18, 22, 39]. The nested PCR assay enhances the sensitivity and/or specificity of PCR amplification [40]. It consists of two sequential amplification steps, each with a separate set of primers [38, 40]. The binding of two different sets of primers to the same target template improves reaction specificity [40]. Therefore, differentiation and identification of megalocytiviruses can be achieved by amplifying a particular viral gene using PCR [41].

The gill and mucus swab samples demonstrated similar positive outcomes (six out of seven swab samples) for *Megalocytivirus*. In this study, an alternative approach to detect the DNA of *Megalocytivirus* without sacrificing the broodstock of *P. scalare* was developed using gill and mucus swabbing. Commonly in aquaculture fish samples, pathogen analysis from visceral organs is performed after sacrificing the fish [42, 43]. Previously, internal organs such as the kidney, spleen, brain, and liver were harvested from ornamental fish, including Cichlidae, Poeciliidae, Cyprinidae, and Osphronemidae, to detect ISKNV infection in India [23]. Other studies in Malaysia [13, 14, 21] sacrificed ornamental fish to detect ISKNV by PCR. However, this lethal sampling method is inappropriate for irreplaceable broodstocks [42]. Therefore, non-lethal sampling techniques such as the swab method are highly recommended. Zainathan *et al*. [44] evaluated the use of swabs and organs to detect the Tasmanian salmon reovirus. The results demonstrated that swabs were as effective as organ samples. Mucus swabs were previously used in a Chinese study on crucian carp [45]. A positive result indicated *Carassius auratus* herpes virus DNA could be detected using the mucus swab sampling method. To detect *Megalocytivirus*, gill and mucus swab samples were examined, as *Megalocytivirus* can cause mucus hypersecretion [46], and high viral concentrations of ISKNV have been demonstrated in fish gills [47]. The use of swab sample collection is inexpensive, less time-consuming [45], able to sample a large population of uncommon fish species without reducing their numbers, allows repeated sampling from a single specimen, and sustains animal welfare by avoiding death in research [48]. Therefore, it is worth mentioning that the swab method is strongly suggested for the detection of *Megalocytivirus* DNA at the farm.

The sequencing analysis of MCP gene of *Megalocytivirus-*positive samples in this study indicated high sequence identity to ISKNV from Asian countries, at 98% and 99% similarity. This result agrees with those of other studies that have been conducted to detect the presence of *Megalocytivirus* in ornamental fish from Malaysia [13, 14, 21] and other countries, such as India [23], Thailand [18], Brazil [22], and Germany [39]. These studies also revealed high similarities (100%) with the ISKNV isolates of Asian and Australian origin. According to Nolan *et al*. [37], several species of ornamental fish such as platys (*X. maculatus*), steel blue killifish (*Atriplex gardneri*), balloon mollies (*Poecilia latipinna*), red tux swordtails (*X. helleri*), and red tiger oscars (*Aetobatus ocellatus*) imported from Singapore, Sri Lanka, and Malaysia collected from quarantine approved premises at Australia’s border control, revealed that it shared 100% sequence identity with *Megalocytivirus* Sabah/RAA1/2012 strain BMGIV48 MCP gene (GenBank accession number JQ253374.1). Phylogenetic analysis showed that all positive samples from Asian countries in this study belonged to the ISKNV genotype clade I. This result is also supported by previous studies by Zainathan *et al*. [13], Zainathan *et al*. [14], and Zainathan *et al*. [21] in Peninsular Malaysia, which demonstrated that all *Megalocytivirus*-positive ornamental fish specimens clustered as ISKNV genotype I.

All water samples in this study were positive for *Megalocytivirus*. This result provided evidence of horizontal transmission of *Megalocytivirus* [36, 49]. Untreated and/or contaminated water can cause *Megalocytivirus* transmission (reviewed in [8]). Previously, the *Megalocytivirus* strain RSIV was shown to spread via seawater, which is considered the main route of virus transmission [50]. The infectivity of RSIV can be maintained for approximately a week in environmental seawater at 15°C [51]. Similarly, the ISKNV can be transmitted via contaminated water from an asymptomatic carrier [36]. Vertical transmission of *Megalocytivirus* in *P. scalare* was not detected in this study to avoid broodstock scarification. Vertical transmission of *Megalocytivirus* from the infected broodstock of ornamental fish to eggs and sperm has not been proven [9]. However, a recent study was conducted on *Iridovirus* infections among groupers (*Epinephelus* spp.) cultured on the Seribu Islands, Indonesia [52]. Histopathological analysis demonstrated inclusion body bearing and inflammation in the grouper gonads. This result indicated that the transmission of *Iridovirus* could be vertical.

The *Megalocytivirus*-associated risk factors identified in this study included hatchery management, water quality parameters (temperature, DO, pH, nitrite, nitrite, and ammonia), water and sediment samples, live feed, snails, wild fish, and life stage. Based on this study*, P. scalare* is more susceptible to *Megalocytivirus* at high temperatures (with a positive correlation between temperature and *Megalocytivirus* incidence). Similarly, a higher number of water samples tested positive for *Megalocytivirus* DNA at higher temperatures. Water temperature is the most crucial factor influencing the presence of viruses in aquaculture [53]. *Megalocytivirus* multiplication is enhanced by high water temperatures between 20°C and 32°C [8]. DGIV was shown to be able to infect Murray cod cohabited with infected dwarf gourami (*T. lalius*) at 27°C [8]. Limited studies have reported the survivability of *Megalocytivirus* using other water quality parameters, such as nitrate, nitrite, DO, and pH in ornamental fish, water, and sediment. However, suboptimal water quality conditions promote pathogen proliferation in farmed fish and their habitat [54]. Viral infections can be reduced by regularly monitoring physicochemical factors (DO, nitrate, nitrite, and ammonia) [55]. A previous study investigated the relationship between water physicochemical parameters and the presence of pathogens in marine cage-cultured fish [56]. The results of canonical correspondence analysis and principal component analysis demonstrated a negative correlation between nitrite (0.0030–0.0100 mg/L) and the presence of *Iridovirus* in groupers due to fluctuating water physicochemical properties [56]. In a study conducted on white spot syndrome virus (WSSV), Xue *et al*. [57] stated that the replication of WSSV in shrimp was not affected by ammonia concentration, and the relationship between WSSV propagation and total ammonia concentration requires further study.

Other possible risk factors that could increase the number of positive samples for *Megalocytivirus* in the ornamental fish farms include snails, sediment, live feed (*Moina* spp. and *tubifex* worms), and wild fish. According to Hoque *et al*. [58], many aquaculturists have reported economic losses due to snails in ponds. Snails can carry viruses or viral DNA to other ponds or parts of a farm. In this study, snails were collected and screened for *Megalocytivirus* DNA because of their large numbers at the farm. Similarly, two golden apple snails (*Pamacea canaliculata*) collected from an aquaculture farm in China were positive for the presence of decapod iridescent virus 1 in farmed white-leg shrimp, *Penaeus vannamei* [59]. *Megalocytivirus* infections in freshwater snails have not yet been reported. However, the presence of *Megalocytivirus* DNA in snails in this study could be one of the routes of *Megalocytivirus* transmission on this farm. Therefore, common methods to control snail populations can be applied at farms, such as using banana leaves, coconut leaves, palm leaves, and bamboo baskets in ponds for 15–20 days as substrates for snails [58, 60]. To date, no studies have reported the viability of *Megalocytivirus* DNA in sediment samples. However, viruses from genus *Ranavirus* belonging to the same family as *Megalocytivirus* (*Iridoviridae*) can survive in sediments for months [61]. According to Brunner *et al*. [62], ranaviruses can be transmitted horizontally through soil. Similarly, FV3 has been shown to survive in untreated sediment from England; nevertheless, the survivability of the virus was low owing to high water temperature [63]. The study showed that 90% viral titer in untreated sediment was reduced at 30°C per day compared to 10 days at 4°C.

Both live feed samples of *Tubifex* worms and *Moina* spp. were positive for the presence of *Megalocyytivirus*. *Tubifex* worms and *Moina* spp. were used to feed the *P. scalare* broodstock and juveniles at the farm and were bought directly from the supplier. The Australian Department of Agriculture and Water Resource [64], stated that feed is one of the major routes of disease transmission on farms. Among all types of feed, live feed has a high and significant risk, depending on the pathogen of concern. According to Gorgoglione *et al*. [65], to prevent the disease from being transmitted into ornamental fish (killifish and zebrafish) by feeding, the fish should not feed on infected frozen or live feeds, as it may cause the transfer of the fish pathogens. Hence, strict routine control of live feeds must be implemented to prevent the introduction of pathogens into aquaculture farms. Based on this study, wild fish were found to be one of the risk factors for *Megalocytivirus* incidence. The previous studies by Bajpai *et al*. [36] and Swaminathan *et al*. [46] have reported the occurrence of ISKNV in wild fish, including wild pearlspot (*Etroplus suratensis*) and ISKNV-like viruses in 39 wild fish species from India. The fish stage was shown to be one of the risk factors for the transmission of *Megalocytivirus* in *P*. *scalare*. In contrast, Johan and Zainathan [8] and Subramaniam *et al*. [53] reported that the juvenile stage of ornamental fish is more susceptible to *Megalocytivirus* infections. Juvenile to young adult *T*. *lalius* have been shown to be positive for *Megalocytivirus* [8]. In addition, juvenile *P. scalare* in the United Kingdom and orange chromide cichlids (*Etroplus* *maculatus*) in Canada have been infected with *Megalocytivirus* (reviewed in Johan and Zainathan [8]).

The experimental design followed the farm management routine to determine the source of *Megalocytivirus* infection on the farm. The farm has been operating for the past 38 years. Based on the results, many unintended aspects of husbandry and hatchery management occurred on the farm. Unsatisfactory biosecurity measures and asymptomatic *Megalocytivirus* carriers may lead to horizontal transmission of the virus, which could be a major cause of infection in ornamental fish farms. The lack of awareness of biosecurity measures may cause outbreaks of infectious diseases and huge losses due to mortality cases [66]. Most aquaculture producers practice poor husbandry measures and do not consult veterinarians when using drugs for ornamental fish [67]. Biosecurity measures and good husbandry can minimize pathogenic infection of pathogens [9]. In addition, it was suggested that an enhancement in monitoring, detection, and prevention, as a step towards strengthened biosecurity due to the expansion of the aquaculture industry in Malaysia over the years, could lead to disease outbreaks, thus affecting the economy [9].

## Conclusion

The presence of *Megalocytivirus* in *P. scalare* was demonstrated using nested PCR. All positive samples (n = 312) showed 95%–99% similarity to ISKNV genotype I from Asian countries. The risk factors identified in this study included water parameters, water, live feeds, snails, sediment, wild fish, and life stages of *P. scalare* which are associated with the precipitation of *Megalocytivirus* outbreaks in Malaysian *P. scalare* from ornamental fish farms. Positive water samples indicated that *Megalocytivirus* has been transmitted horizontally. Gill and mucus swab samples can be utilized as non-invasive sample collection methods to detect *Megalocytivirus* in ornamental fish. Moreover, it can be concluded that inadequate biosecurity and aquaculture husbandry techniques are the primary causes of *Megalocytivirus* outbreaks on ornamental fish farms. The findings of this study may contribute to a better understanding of *Megalocytivirus* risk factors and epidemiology in Malaysia. These findings emphasize the importance of effective biosecurity measures in the ornamental fish industry. Examples include the execution of moderate and inexpensive biosecurity measures, adequate water filtration systems, human movement limitations within hatcheries, and adherence to sanitization guidelines. Furthermore, problem control and prevention procedures based on locally applicable strategies and globally accepted principles are recommended. Strategies should concentrate on preventing the spread of infection rather than treating diseased stocks. Surveillance is helpful for the early identification of disease etiological agents, in addition to detecting current infectious agents and their widespread distribution in aquaculture and wild reservoirs. Another advantage of surveillance is the identification of the epidemiological patterns of viral diseases that are endemic to different ecosystems. In addition, it can be used to locate endemic hotspots and antigenic variations that direct vaccine development. Moreover, substantial genetic analysis is needed to fully understand the epidemiology of *Megalocytivirus* and determine the variation in *Megalocytivirus*-infected ornamental fish in Malaysia for future vaccine development. Metagenomics is a useful tool for the discovery of novel viral strains by analyzing new sequences that do not share homology with sequences in reference databases. Therefore, metagenomic analyses will inevitably hasten the identification of new infectious agents in aquaculture. In addition, it can be used to ascertain the viral population in fish farming-related aquatic environments.

## Authors’ Contributions

SCZ, MDDA, and SNESJF: Supervised the study and edited the manuscript. CACJ: Conducted laboratory work and analyzed the data. SCZ, MDDA, SNE, and CACJ: Performed statistical analyses. SCZ and CACJ: Drafted the manuscript. All the authors have read, reviewed, and approved the final version of the manuscript.
